# NOVEL SERUM BIOMARKERS FOR SARCOPENIA: INSIGHTS FROM CLINICAL DATA AND STUDIES USING C2C12 MYOBLASTS

**DOI:** 10.1093/geroni/igae098.3679

**Published:** 2024-12-31

**Authors:** Hsien-Hao Huang

**Affiliations:** Taipei Veterans General Hospital, Taipei, Taipei, Taiwan (Republic of China)

## Abstract

**Introduction:**

Sarcopenia, the progressive loss of skeletal muscle mass and function, is a critical public health issue among older adults with underlying functional decline and increased risk of adverse health outcomes. While roles have been established for gut hormones in age-associated anorexia, their direct influence on sarcopenia remains unknown. We evaluated the associations between elevated glucagon-like peptide-1 (GLP-1) and cholecystokinin (CCK) serum levels and sarcopenia and investigated their direct effects on myogenesis and muscle fusion.

**Methods:**

Total 179 patients with sarcopenia were enrolled in this study. This study utilized clinical biomarkers, Bioelectrical Impedance Analysis (BIA), and comprehensive geriatric assessment. Concurrently, C2C12 myoblasts were treated with varying concentrations of GLP-1 and CCK (0, 1, 10, and 100 ng/mL) to examine their influence on myoblast differentiation and myocyte fusion, as assessed by myosin heavy chain (MYH1/2) expression using western blot, and fusion index using immunostaining. Results Patients with sarcopenia exhibit significantly elevated levels of GLP-1 and CCK. GLP-1 impedes the differentiation of myoblasts into mature myocytes in vitro, thereby potentially restricting myogenesis, and GLP-1 treatment negatively impacts myocyte fusion with myotubes. In contrast, CCK inhibits myocyte fusion, a crucial step in muscle formation, without altering the initial differentiation of myoblasts. These findings underscore the critical role of these hormones in muscle development and provide novel insights into their contribution to sarcopenia.

**Conclusion:**

This study supports the use of GLP-1 and CCK as key biomarkers for sarcopenia, suggesting that their impact extends beyond metabolism and directly affects muscle integrity.

